# Statin-Associated Immune-Mediated Necrotizing Myopathy Presenting as Persistent Hypertransaminasemia

**DOI:** 10.7759/cureus.100987

**Published:** 2026-01-07

**Authors:** Rita Bragança, Nuno Ferreira da Silva, Elisa Macedo Brás

**Affiliations:** 1 Internal Medicine, Unidade Local de Saúde de Trás-os-Montes e Alto Douro, Vila Real, PRT

**Keywords:** antibodies, hepatitis b virus, hydroxymethylglutaryl-coa reductase inhibitors, myositis, transaminases

## Abstract

Immune-mediated necrotizing myopathy is an uncommon autoimmune muscle disease that can be triggered by statin exposure and is often associated with antibodies against 3-hydroxy-3-methylglutaryl-coenzyme A reductase (HMGCR). Typical presentations include subacute, symmetric proximal weakness with marked elevation of muscle enzymes, but some patients come to medical attention only because of abnormal laboratory tests, which can delay recognition. We report a middle-aged man with dyslipidemia and chronic hepatitis B virus infection who was referred for persistent elevation of aminotransferases on routine follow-up. Despite minimal muscle complaints, evaluation revealed marked hyperCKemia, strongly positive anti-HMGCR antibodies, and a muscle biopsy consistent with pauci-inflammatory necrotizing myopathy. Statins were discontinued, and combined glucocorticoid-methotrexate therapy led to progressive clinical improvement and complete biochemical remission. This case underscores the importance of considering an underlying myopathy in patients with otherwise unexplained hypertransaminasemia, particularly in the setting of statin exposure and coexisting liver disease.

## Introduction

Immune-mediated necrotizing myopathy (IMNM) is a rare and severe condition that represents a distinct entity within the spectrum of idiopathic inflammatory myopathies [1].

Most IMNMs are linked to myositis-specific autoantibodies against the signal recognition particle (anti-SRP) or 3-hydroxy-3-methylglutaryl-CoA reductase (anti-HMGCR), although approximately 20% of patients with IMNM are seronegative [2].

Recent population-based data from cohorts in Greater Manchester (UK) and South Australia (Australia) estimated the incidence of anti-HMGCR IMNM to range from 0.9 to 2.4 cases per million population per year between 2018 and 2022 [3].

Most patients with anti-HMGCR myopathy share a characteristic phenotype, with proximal muscle weakness, markedly elevated creatine kinase (CK), myopathic changes on electromyography (EMG), and magnetic resonance imaging (MRI) showing widespread thigh muscle edema, typically with prominent involvement of the anterior compartment [4,5].

Anti-HMGCR IMNM is strongly associated with prior or current statin use and predominantly affects middle-aged or older adults [6]. In contrast to self-limited toxic statin myopathy, this condition typically persists or progresses despite statin withdrawal and often requires high-dose glucocorticoids combined with additional immunosuppressive agents [7].

We present the case of a statin-treated adult in whom persistent hypertransaminasemia and subtle muscle weakness ultimately led to the diagnosis of anti-HMGCR IMNM.

This clinical case was previously presented as a meeting abstract at the 18th Meeting of the Internal Medicine Residents Working Group (Núcleo de Internos de Medicina Interna) in Portugal.

## Case presentation

A 61-year-old man was referred to the Internal Medicine clinic for the evaluation of abnormal liver biochemistry detected on routine follow-up. His medical history included dyslipidemia, essential hypertension, asthma, and chronic hepatitis B virus (HBV) infection (hepatitis B e antigen (HbeAg) negative). He had no relevant family history of neuromuscular or autoimmune disease.

He described the onset of progressive asthenia in late 2023, after starting atorvastatin 10 mg once daily for dyslipidemia. Over the following months, he noted worsening fatigability in the lower limbs, with difficulty climbing stairs and performing exertional activities. Because of progressive asthenia and a rise in CK (CK 3,097 U/L; upper limit of normal (ULN) <190 U/L), atorvastatin was discontinued in June 2024 by his general practitioner, with partial symptomatic improvement. He was briefly switched to rosuvastatin 5 mg, which was also stopped when symptoms persisted. At the time of referral, he remained off statin therapy.

Over approximately six months, he reported unintentional weight loss of about 5 kg (from 82 kg to 77 kg). He denied fever or other constitutional symptoms.

He denied myalgias, muscle cramps, arthralgias, or morning stiffness; Raynaud phenomenon or photosensitivity; oral or genital ulceration, xerostomia, or xerophthalmia; esophageal symptoms including dysphagia; abdominal pain or other gastrointestinal symptoms (including changes in bowel habits); and dark urine, pale stools, or pruritus.

On examination, he was afebrile and hemodynamically stable. There were no stigmata of chronic liver disease, lymphadenopathy, digital ulcers, skin thickening, rashes, or telangiectasias. Cardiopulmonary and abdominal examinations were unremarkable, with no hepatosplenomegaly. Neurologic examination revealed intact cranial nerves, normal muscle tone and deep tendon reflexes, preserved sensation, and mild symmetrical weakness in both proximal and distal muscle groups of all four limbs (Medical Research Council Muscle Scale 4+/5). Gait remained normal, and there was no Gowers' sign.

Initial laboratory testing showed a hepatocellular pattern of liver enzyme elevation (R-factor of 7.6), with moderately increased aspartate aminotransferase (AST) and alanine aminotransferase (ALT), while alkaline phosphatase, gamma-glutamyl transferase, total bilirubin, albumin, and coagulation parameters remained within reference ranges. Renal function and serum electrolytes, including calcium, magnesium, potassium, and sodium, were within the respective reference ranges. In contrast, muscle enzymes were markedly elevated, with pronounced hyperCKemia and concomitant increases in creatine kinase MB isoenzyme (CK-MB), myoglobin, and aldolase. C-reactive protein and complete blood count were within reference ranges (Table 1).

**Table 1:** Baseline liver and muscle enzyme profile ALT: alanine aminotransferase; AST: aspartate aminotransferase; CK: creatine kinase; CK-MB: creatine kinase MB isoenzyme; GGT: gamma-glutamyl transferase

Test	Result at presentation	Reference range
ALT	219	<41 U/L
Albumin	4.4	3.4-4.8 g/dL
Aldolase	26.9	<7.6 U/L
Alkaline phosphatase	91	40-130 U/L
AST	173	<40 U/L
CK	3,097	<190 U/L
CK-MB isoenzyme	95.5	<4.9 ng/mL
GGT	33	10-49 U/L
International normalized ratio	1.02	<1.2
Myoglobin	709	<72 ng/mL
Total bilirubin	0.8	<1.2 mg/dL

The patient had evidence of HbeAg-negative chronic HBV infection, and HBV DNA by polymerase chain reaction (PCR) was undetectable, ruling out a significant contribution of active HBV replication to the abnormal liver biochemistry.

Given the pattern of hepatocellular enzyme elevation with normal cholestatic markers and the marked hyperCKemia, a myopathic rather than primary hepatic source of AST and ALT was suspected. Nevertheless, primary hepatic causes were also systematically excluded (Table 2), and abdominal ultrasonography was unremarkable, showing no structural liver or biliary abnormalities.

**Table 2:** Viral serology and metabolic workup Ab: antibody; Ag: antigen; anti-HBc: hepatitis B core antibody; anti-HBs: hepatitis B surface antibody; anti-HBe: hepatitis B e antibody; HAV: hepatitis A virus; CMV: cytomegalovirus; EBV: Epstein-Barr virus; HBsAg: hepatitis B surface antigen; HBeAg: hepatitis B e antigen; HBV: hepatitis B virus; HCV: hepatitis C virus; HIV: human immunodeficiency virus; IgA: immunoglobulin A; IgG: immunoglobulin G; IgM: immunoglobulin M; IGRA: interferon-gamma release assay; PCR: polymerase chain reaction; TIBC: total iron-binding capacity

Test	Result	Reference range
Serology		
Anti-HAV IgM	Non-reactive	Non-reactive/reactive
Anti-HAV total	Reactive	Non-reactive/reactive
Anti-HBc total	Reactive	Non-reactive/reactive
Anti-HBe	Reactive	Non-reactive/reactive
Anti-HBs	Non-reactive	Non-reactive/reactive
Anti-HCV	Non-reactive	Non-reactive/reactive
CMV IgG+IgM	Non-reactive	Non-reactive/reactive
EBV IgG	Reactive	Non-reactive/reactive
EBV IgM	Non-reactive	Non-reactive/reactive
HBeAg	Non-reactive	Non-reactive/reactive
HBsAg	Reactive	Non-reactive/reactive
HIV 1/2 Ag/Ab combo	Non-reactive	Non-reactive/reactive
Syphilis IgG+IgM	Non-reactive	Non-reactive/reactive
Biochemistry/metabolism		
Ceruloplasmin	22.3	20-60 mg/dL
Copper	15.05	8.8-17.5 ug/mL
IgA	910	600-1570 mg/dL
IgG	271	50-373 mg/dL
IgM	101	40-325 mg/dL
Serum iron	64	37-145 µg/mL
Serum protein electrophoresis	No monoclonal peaks	-
TIBC	164	228-360 mg/dL
Transferrin saturation	39	>20%
Other		
HBV DNA (PCR, plasma)	Not detectable	Not detectable
IGRA	Negative	Negative

An extended myositis antibody immunoblot was performed and was negative. A second, extended myositis panel performed at a reference center confirmed these negative results but identified high-titer anti-HMGCR antibodies (Table 3).

**Table 3:** Autoimmune serologic workup cN-1A: cytosolic 5′-nucleotidase 1A; dsDNA: double-stranded deoxyribonucleic acid; EJ: glycyl-tRNA synthetase antibody; ENA: extractable nuclear antigens; HMGCR: 3-hydroxy-3-methylglutaryl-coenzyme A reductase; Ku: Ku antigen; LKM: liver-kidney microsomal; MDA5: melanoma differentiation-associated gene 5; Mi-2: chromodomain-helicase-DNA-binding protein 4; NOR90: nucleolar organizer region 90; NXP2: nuclear matrix protein 2; OJ: isoleucyl-tRNA synthetase antibody; PCNA: proliferating cell nuclear antigen; PDGFR: platelet-derived growth factor receptor; PL-7: threonyl-tRNA synthetase antibody; PL-12: alanyl-tRNA synthetase antibody; PM-Scl: polymyositis/scleroderma overlap antigen; RNP: ribonucleoprotein; RNA Pol III: RNA polymerase III; Scl-70: DNA topoisomerase I; SAE1: SUMO-activating enzyme subunit 1; Sm: Smith antigen; SRP: signal recognition particle; SS-A/Ro: Sjögren's syndrome-related antigen A; SS-B/La: Sjögren's syndrome-related antigen B; TIF1γ: transcription intermediary factor 1-gamma; UQ: units of quantification

Test	Result	Reference range
Indirect immunofluorescence		
Anti-HMGCR (quantitative)	172.4 UQ	<20 UQ
Anti-LKM antibodies	<1:20	<1:20
Anti-mitochondrial antibodies	<1:80	<1:80
Anti-smooth muscle antibodies	<1:40	<1:40
Antinuclear antibodies	<1:40	<1:40
Fluorescence enzyme immunoassay		
ENA screen (dsDNA, SS-A/Ro, SS-B/La, Sm, RNP, Scl-70, centromere, Jo-1, fibrillarin, RNA Pol III, PM-Scl, PCNA, Mi-2, ribosomal P)	Negative	Negative
Immunoblot		
Anti-centromere A	Negative	Negative
Anti-centromere B	Negative	Negative
Anti-fibrillarin	Negative	Negative
Anti-Ku	Negative	Negative
Anti-NOR90	Negative	Negative
Anti-PDGFR	Negative	Negative
Anti-PM-Scl 100	Negative	Negative
Anti-PM-Scl 75	Negative	Negative
Anti-RNA polymerase III	Negative	Negative
Anti-RNA polymerase III	Negative	Negative
Anti-Ro52	Negative	Negative
Anti-Scl-70	Negative	Negative
Anti-SRP	Negative	Negative
Anti-Th/To	Borderline	Negative
cN-1A	Negative	Negative
Jo-1, PL-7, PL-12, EJ, OJ	Negative	Negative
Mi-2α, Mi-2β, TIF1γ, MDA5, NXP2, SAE1	Negative	Negative

**Figure 1:**
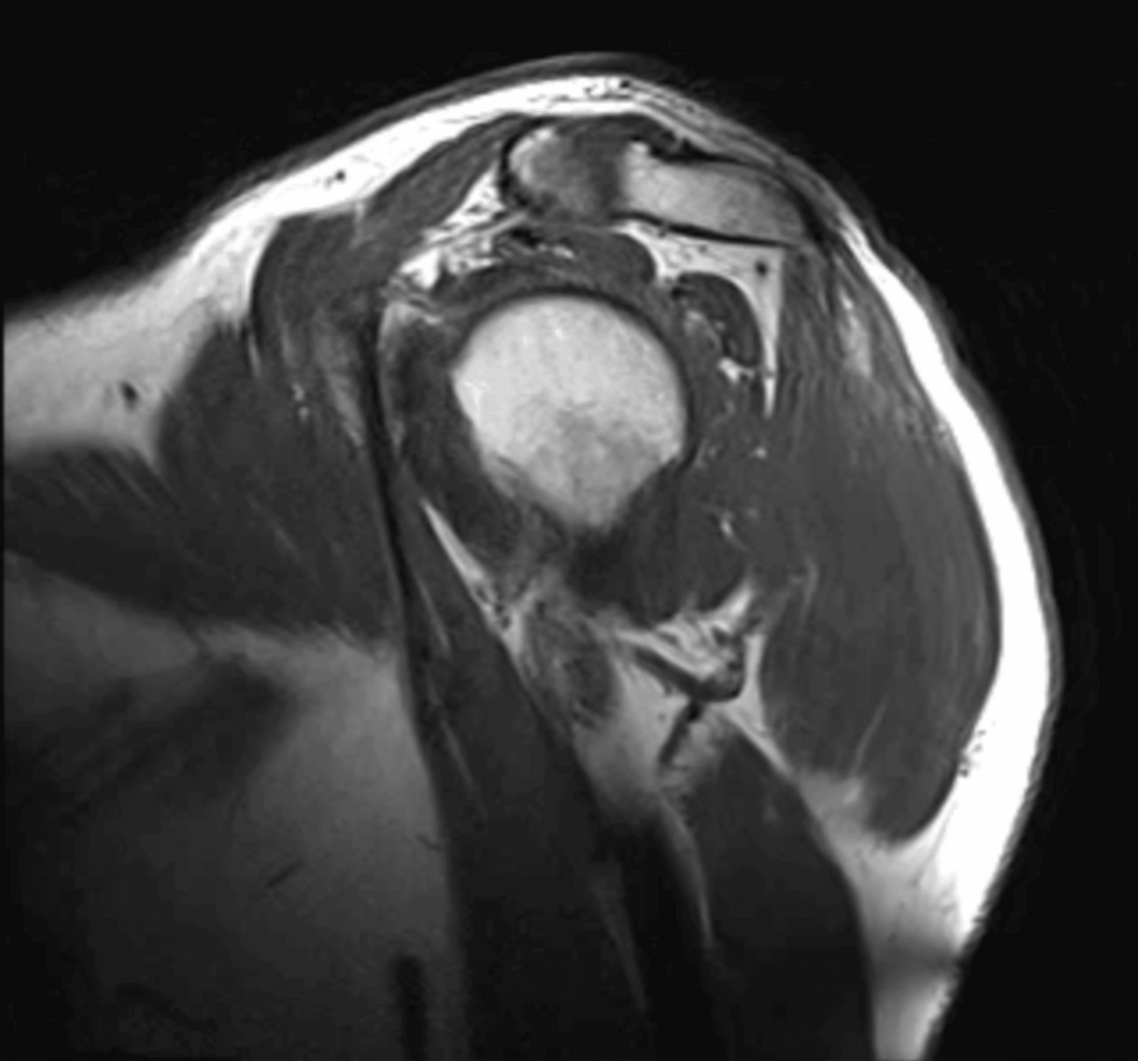
Sagittal T1-weighted magnetic resonance image of the shoulder Sagittal T1-weighted magnetic resonance image of the shoulder girdle demonstrating preserved muscle bulk and homogeneous signal of the peri-scapular and peri-humeral muscles, without edema, fatty replacement, or focal lesions, consistent with absence of active myositis or chronic myopathic changes.

To assess muscle involvement, MRI of the proximal upper and lower limb muscles was performed and showed no edema, fatty replacement, or other abnormalities suggestive of active myositis or chronic myopathy (Figure 1). However, electrophysiologic assessment with nerve conduction studies and concentric needle EMG of the right biceps brachii, common extensor digitorum, iliopsoas, tibialis anterior, and vastus medialis showed rare fibrillation potentials and positive sharp waves in the iliopsoas and biceps, with voluntary activation characterized by polyphasic motor unit potentials of increased amplitude and duration in these proximal muscles. The remaining sampled muscles were unremarkable. The overall impression was of a myopathic process affecting proximal muscle groups of both upper and lower limbs, with scant signs of active denervation compatible with ongoing muscle fiber injury.

A deltoid muscle biopsy was performed. Histopathologic examination demonstrated focal sarcolemmal upregulation of major histocompatibility complex (MHC) class I on non-necrotic fibers. There were scattered regenerating fibers but no significant endomysial or perimysial inflammatory infiltrate, no vasculitis, no rimmed vacuoles, and no features of muscular dystrophy. The overall picture was consistent with a pauci-inflammatory necrotizing myopathy in the appropriate clinical and serologic context.

Given the patient's age and recent onset of systemic symptoms, an occult neoplasm was considered, so we performed an age-appropriate occult malignancy workup. Thoracoabdominopelvic computed tomography was unremarkable. Upper and lower gastrointestinal endoscopy did not reveal malignancy.

Pulmonary function tests and diffusing capacity of the lung for carbon monoxide were within normal limits, ruling out clinically relevant pulmonary involvement.

Considering the subacute asthenia and exertional fatigability, marked hyperCKemia and elevated aldolase and myoglobin, strongly positive anti-HMGCR antibodies, EMG evidence of proximal myopathy with active denervation, and exclusion of alternative etiologies, a diagnosis of statin-associated anti-HMGCR IMNM was established.

Immunosuppressive therapy was initiated with oral prednisolone 40 mg once daily (approximately 0.5 mg/kg) and methotrexate 10 mg once weekly, uptitrated to 15 mg once weekly as tolerated, as a steroid-sparing agent. Given his chronic hepatitis B, antiviral prophylaxis for reactivation with entecavir 0.5 mg once daily was started, with concurrent periodic HBV DNA monitoring.

**Figure 2:**
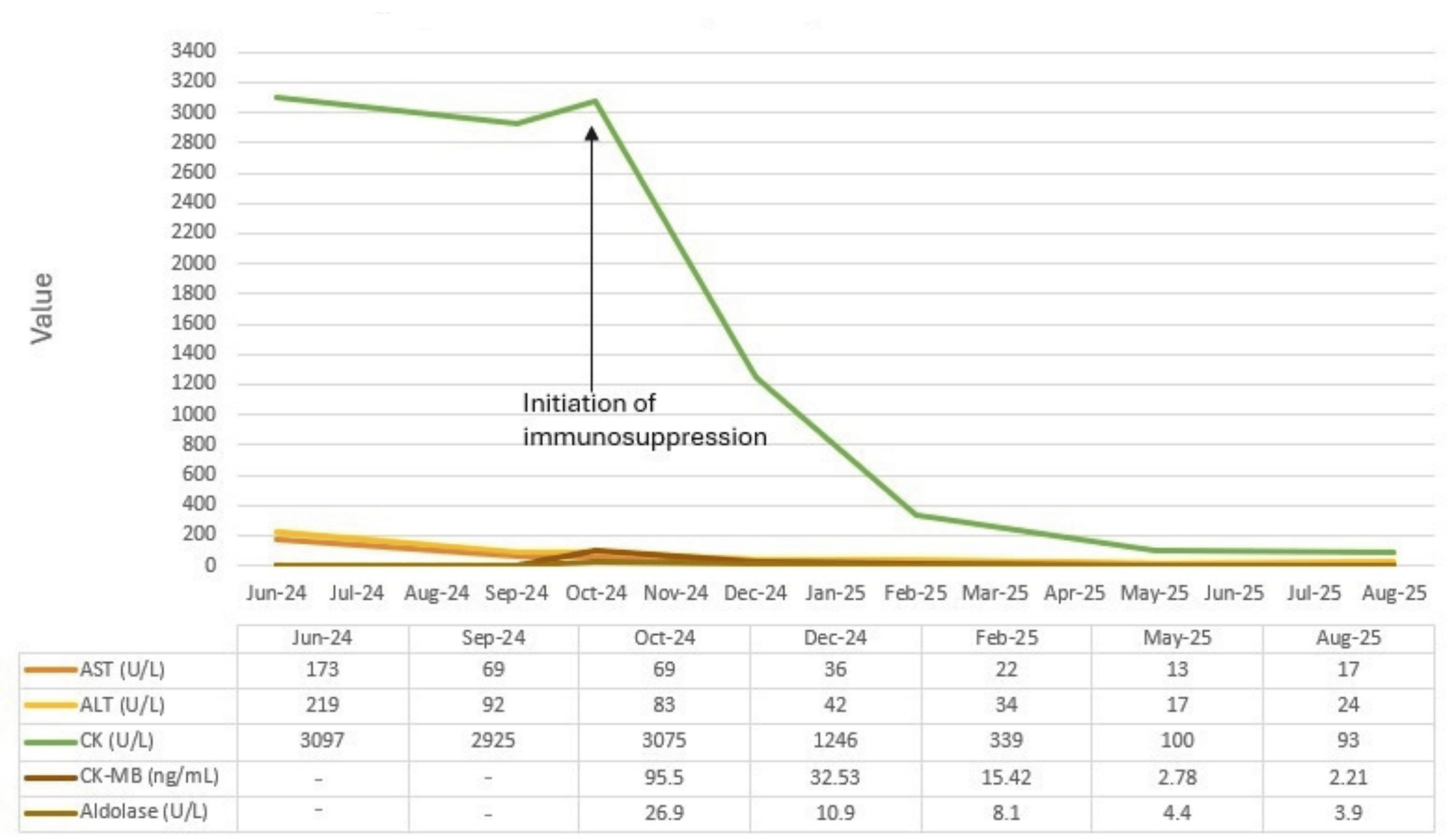
Temporal evolution of AST, ALT, CK, CK-MB, and aldolase Reference ranges: AST <40 U/L, ALT <41 U/L, CK <190 U/L, CK-MB <4.9 ng/mL, and aldolase <7.6 U/L. AST: aspartate aminotransferase; ALT: alanine aminotransferase; CK: creatine kinase; CK-MB: creatine kinase MB isoenzyme

Over subsequent months, the patient reported progressive improvement in energy and functional capacity, without the development of new systemic manifestations. Serial laboratory tests documented the normalization of AST and ALT and a steady decline in CK, CK-MB, and aldolase, as illustrated in Figure 2.

At the last follow-up, he was asymptomatic, having completed a six-month taper of glucocorticoids, remained on maintenance methotrexate, and had experienced no relapses.

## Discussion

This case highlights several important aspects of anti-HMGCR IMNM and underscores diagnostic pitfalls when liver and muscle injury coexist.

Most patients with anti-HMGCR IMNM present with subacute, often severe, symmetrical proximal weakness and markedly elevated CK [2]. Our patient complained primarily of fatigue and lower limb "tiredness" with a slight decrease in muscular strength on manual testing. Similar paucisymptomatic or mild phenotypes have been described, particularly in older individuals, who may respond better to standard immunosuppression than younger patients [8].

The absence of myalgia and overt weakness likely contributed to the initial hepatic focus of the investigation.

Persistent hypertransaminasemia was an early clue to underlying muscle disease. AST and ALT are not liver-specific; skeletal muscle injury can cause substantial aminotransferase elevations, particularly in rhabdomyolysis and inflammatory myopathies [9]. Several series have emphasized that unrecognized muscle disease is an important non-hepatic cause of elevated transaminases and that the diagnostic delay may be long if CK is not measured [9-11]. In our patient, the pattern of AST and ALT elevation with normal alkaline phosphatase and gamma-glutamyl transferase, combined with a CK >3,000 U/L, strongly suggested a muscular rather than hepatic source, despite the background of chronic HBV infection. This underscores the importance of routine CK measurement in patients with otherwise unexplained aminotransferase elevations, especially when cholestatic markers and liver imaging are unremarkable.

The serologic profile was highly informative. High-titer anti-HMGCR antibodies have emerged as a sensitive and specific biomarker for anti-HMGCR IMNM, and current classifications allow the diagnosis of IMNM based on compatible clinical features, elevated CK, and these antibodies, even when muscle biopsy is not classic [2]. In our patient, repeated broad myositis panels were negative, but the strongly positive anti-HMGCR result, together with the clinical and biochemical features, was decisive. The borderline anti-Th/To result, in the absence of Raynaud phenomenon, sclerodactyly, telangiectasias, or interstitial lung disease, was considered an incidental finding.

The histopathologic findings, though not florid, were compatible with IMNM. Classic features include scattered necrotic and regenerating fibers with macrophage-rich infiltrates and diffuse MHC class I upregulation, often with minimal lymphocytic inflammation [1,2]. Our patient's biopsy showed focal MHC class I expression and sparse regenerating fibers without overt necrosis or inflammation, underscoring that IMNM lesions may be patchy or subclinical at the time of sampling. In such cases, serology and EMG findings become even more critical.

This case reinforces the well-established association between statin exposure and anti-HMGCR IMNM. Statins upregulate HMGCR expression in muscle, potentially enhancing autoantigen availability and breaking tolerance in genetically predisposed individuals [2-4]. Unlike self-limited statin-associated muscle symptoms, toxic statin myopathy, that usually develop early and improve within ~2-4 weeks after statin discontinuation, anti-HMGCR IMNM reflects a delayed immune-mediated process and may occur months to years after statin initiation, persisting (or even progressing) despite statin withdrawal. Clinically, this distinction is helpful: toxic statin myopathy is more often dominated by myalgias and improves with stopping the drug, whereas anti-HMGCR IMNM is characterized by objective weakness with ongoing CK elevation and typically requires immunosuppressive treatment [12]. Once autoimmunity is established, disease activity usually persists despite statin withdrawal and requires immunosuppression [13].

Our patient had been exposed to two statins (atorvastatin and rosuvastatin) and improved only after both discontinuation and initiation of glucocorticoids plus methotrexate.

Overall, this case illustrates how a liver-focused referral can uncover an autoimmune myopathy when a systematic, multidisciplinary approach is applied. It also emphasizes that normal muscle MRI and minimal biopsy changes do not exclude IMNM when clinical, biochemical, and serologic evidence is strong.

## Conclusions

This case illustrates that anti-HMGCR IMNM can present in a subtle and misleading fashion, with persistent elevation of aminotransferases as the dominant abnormality in a patient with coexisting liver disease. The combination of hepatocellular enzyme elevation, normal cholestatic markers, and marked hyperCKemia was crucial in redirecting the workup from a purely hepatic focus toward an underlying myopathic process. Our findings reinforce the importance of routinely measuring CK in patients with otherwise unexplained hypertransaminasemia, particularly in the context of statin exposure, and of considering IMNM even when muscle symptoms are mild and imaging or biopsy findings are not florid.

In addition, this case highlights the central role of myositis-specific autoantibodies, especially anti-HMGCR, in establishing the diagnosis, as well as the value of integrating serology with EMG and targeted muscle biopsy. Early withdrawal of statins and timely initiation of combined immunosuppressive therapy led to complete clinical and biochemical remission. Clinicians should maintain a high index of suspicion for statin-associated anti-HMGCR IMNM in similar scenarios, as prompt recognition and treatment are essential to prevent progression and long-term disability.
